# Construction of a risk scoring system using clinical factors and *RYR2* polymorphisms for bleeding complications in patients on direct oral anticoagulants

**DOI:** 10.3389/fphar.2023.1290785

**Published:** 2023-11-15

**Authors:** Eun Jeong Jang, Jung Sun Kim, Seo A. Choi, Jeong Yee, Tae-Jin Song, Junbeom Park, Hye Sun Gwak

**Affiliations:** ^1^ College of Pharmacy and Graduate School of Pharmaceutical Sciences, Ewha Womans University, Seoul, Republic of Korea; ^2^ Department of Neurology, Ewha Womans University Seoul Hospital, Ewha Womans University College of Medicine, Seoul, Republic of Korea; ^3^ Division of Cardiology, Department of Internal Medicine, Ewha Womans University Mokdong Hospital, Ewha Womans University College of Medicine, Seoul, Republic of Korea

**Keywords:** direct oral anticoagulants, bleeding, ryanodine receptors, pharmacogenomics, gene polymorphism

## Abstract

**Introduction:** Bleeding is one of the most undesirable complications of direct oral anticoagulants (DOACs). While the ryanodine receptor (*RYR2*) has been related to cardiac diseases, research on bleeding complications is lacking. This study aimed to elucidate the association between *RYR2* and bleeding risk to develop the risk scoring system in patients treated with DOACs.

**Methods:** This study was a retrospective analysis of prospectively collected samples. We selected ten SNPs within the *RYR2* gene, and two models were constructed (Model I: demographic factors only, Model II: demographic and genetic factors) in multivariable analysis. Independent risk factors for bleeding were used to develop a risk scoring system.

**Results:** A total of 447 patients were included, and 49 experienced either major bleeding or clinically relevant non-major bleeding. In Model I, patients using rivaroxaban and experiencing anemia exhibited an increased bleeding risk after adjusting for covariates. Upon incorporating genetic factors into Model I, a significant association with bleeding was also observed in cases of overdosing on DOACs and in patients with a creatinine clearance (CrCl) < 30 mL/min, in addition to rivaroxaban and anemia (Model II). Among genetic factors, *RYR2* rs12594 GG, rs17682073 AA, rs3766871 GG, and rs6678625 T alleles were associated with bleeding complications. The area under the receiver operating characteristic curve (AUROC) of Model I was 0.670, whereas that of Model II increased to 0.803, demonstrating better performance with the inclusion of genetic factors. Using the significant variables in Model II, a risk scoring system was constructed. The predicted bleeding risks for scores of 0, 1–2, 3–4, 5–6, 7–8, and 9–10 points were 0%, 1.2%, 4.6%, 15.7%, 41.7%, and 73.3%, respectively.

**Conclusion:** This study revealed an association between *RYR2* and bleeding complications among patients taking DOACs and established a risk scoring system to support individualized DOAC treatment for these patients.

## Introduction

Direct oral anticoagulants (DOACs) are oral anticoagulants that have revolutionized the management of thromboembolic disorders. DOACs have demonstrated comparable efficacy to conventional agents such as coumadin in preventing strokes for patients with atrial fibrillation (AF) and in treating venous thromboembolism (VTE) while exhibiting a reduced risk of bleeding complications (Martin et al., 2016). Unlike warfarin, DOACs exert their effects by directly inhibiting specific clotting factors, including factor Xa or thrombin, without requiring routine monitoring of the international normalized ratio (INR) or dose adjustments. Their rapid onset of action, predictable pharmacokinetics (PKs), and fewer drug-drug interactions collectively position DOACs as an attractive therapeutic alternative to warfarin (Bauer, 2013).

Bleeding is one of the most undesirable complications of anticoagulation treatment (Lip et al., 2018). Although DOACs generally have a safe profile, major bleeding events still occur with a frequency ranging from 3.5% to 5.3% per person-year (Lip et al., 2018). It has also been reported that DOACs may carry risks of gastrointestinal bleeding comparable to or even higher than those associated with warfarin (Feagins and Weideman, 2018). Thus, it is crucial to consider factors that increase the risk of bleeding, such as advanced age, renal impairment, and concomitant use of other medications while on DOAC treatment (Chan et al., 2018). For individuals undergoing anticoagulant treatment, the HAS-BLED score is utilized as a tool to assess the risk of bleeding (Gao et al., 2021). The HAS-BLED encompasses factors such as hypertension, abnormal renal or liver function, stroke, bleeding history or predisposition, an unstable INR, age over 65 years, and the use of drugs or alcohol.

The extent of variability in how individuals respond to DOACs regarding their PKs and pharmacodynamics has not been fully clarified, suggesting the potential involvement of genetic factors. This variability can be understood through gene polymorphisms, such as *CES1* and *ABCB1*, responsible for the activation or transport of DOACs (Raymond et al., 2021; Ferri et al., 2022). In addition, the metabolism of factor Xa inhibitors involves cytochrome P450 (CYP) enzymes, specifically CYP3A4 and CYP3A5 (Raymond et al., 2021). Consequently, prior research has primarily focused on identifying genetic variations within the genes that impact DOAC transport and metabolism (Tseng et al., 2018). Thus, there is a need to identify novel genetic factors associated with DOAC responses.

Ryanodine receptors (RyRs) are Ca^2+^-induced Ca^2+^ release channels that play essential roles in neurons, muscle cells, and epithelial cells. Among three isoforms that encode RyR genes (*RYR*), RYR2 is expressed predominantly in cardiac muscle (Avila et al., 2019), and it has been reported to be associated with cardiac diseases such as arrhythmias and sudden cardiac death (SCD) (Napolitano and Priori, 2007; Steinberg et al., 2023). Abnormalities of perfusion caused by arrhythmias have deleterious effects on hemodynamic instability (Kochiadakis and Kallergis, 2012), which is a significant risk factor for bleeding and mortality (Lee et al., 2013). However, research concerning the impact of *RYR2* on bleeding complications is lacking. Therefore, the objective of this study was to elucidate the association between *RYR* and bleeding risk and to construct the risk scoring system in patients undergoing DOAC treatment.

## Materials and methods

### Study participants and data collection

This retrospective cohort study was conducted at Ewha Womans University Mokdong Hospital and Ewha Womans University Seoul Hospital from June 2018 to December 2021. The study included patients diagnosed with AF who were administered DOACs (namely, apixaban, edoxaban, rivaroxaban, or dabigatran) for a minimum of 3 months. Patients were excluded if they: 1): experienced thromboembolic events during the study period, 2): experienced a bleeding event 1 year after taking DOACs, 3): had minor unverified bleeding events, 4): did not provide a sufficient DNA analysis sample, or 5): withdrew their informed consent. The primary outcome assessed in this study was major bleeding or clinically relevant non-major bleeding (CRNMB), as defined by criteria established by the International Society on Thrombosis and Haemostasis (ISTH) (Schulman et al., 2005; Kaatz et al., 2015). According to the guidelines, cases were considered to have experienced major bleeding if one of the following clinical manifestations was present in the medical chart: 1) fatal bleeding, 2) symptomatic bleeding in a critical organ, or 3) bleeding causing a decrease in hemoglobin level of 20 g/L or more or leading to transfusion of two or more units of white blood or red cells. Patients were recorded as having experienced CRNMB if they had a bleeding history that did not qualify as major bleeding and required any medical or surgical intervention to treat the bleeding.

All procedures were approved by the Ethics Committee of the Institutional Review Board (IRB number: 2019-05-038 and 2018-04-006), and the study adhered to the ethical principles outlined in the 1975 Declaration of Helsinki and its later amendments. Written informed consent was obtained from all participants prior to their enrollment. After obtaining consent, DNA samples were collected prospectively from the blood or saliva of each patient for subsequent DNA analysis. Demographic and clinical information, including sex, age, weight, height, creatinine clearance (CrCl), DOAC prescription data (types, dosage, duration), history of thromboembolic events (stroke, transient ischemic attack, and thromboembolism), bleeding events, comorbidities, smoking and alcohol status, and co-medication, were then retrospectively extracted from the electronic medical records. CYP inhibitors included ritonavir, clarithromycin, quinidine, fluconazole, cyclosporine, amiodarone, dronedarone, diltiazem, verapamil, and imatinib, while CYP inducers included carbamazepine, phenytoin, phenobarbital, and rifampicin. The CHA_2_DS_2_-VASc (congestive heart failure, hypertension, age ≥75 years, diabetes mellitus, stroke, vascular disease, age 65–74 years, and sex category; range 0–9) score for stroke risk and the modified HAS-BLED score for bleeding risk were estimated from their components (Lip et al., 2010; Gao et al., 2021). We used a modified HAS-BLED score that excludes the INR from the calculation since the INR is not monitored in patients taking DOACs (Tsu et al., 2015). A score of ≥3 indicates high risks for CHA_2_DS_2_-VASc and HAS-BLED scores, so we set the cutoff at 3 in the analysis (Lip et al., 2010; Tsu et al., 2015).

### Single nucleotide polymorphism (SNP) selection and genotyping methods

We opted for ten SNPs within the *RYR2* gene and included *ABCB1* rs3842 as a confounder based on previous research findings (Westaway et al., 2011; Akilzhanova et al., 2014; Francia et al., 2015; Chen et al., 2019; Hopton et al., 2022). Comprehensive information regarding these SNPs, including their chromosomal positions, functional information, and wild-type/variant alleles, was obtained from the National Center for Biotechnology Information (Sayers et al., 2022). To assess the minor allele frequency and linkage disequilibrium in Asian populations, Haploreg 4.1 was employed (Ward and Kellis, 2016).

Genomic DNA from blood samples was collected using the QIAamp DNA Blood Mini Kit (QIAGEN GmbH, Hilden, Germany), while saliva samples were analyzed using OraGene-600 kits (DNA Genotek, Ottawa, ON, Canada) and PrepIT reagents (DNA Genotek, OTT, Canada). We employed the TaqMan SNP genotyping assay (Applied Biosystems, Foster City, CA, United States) to genotype eleven SNPs. All SNPs were checked to ensure they were in Hardy–Weinberg equilibrium.

### Statistical analysis

The chi-squared test was utilized to assess genotyping data for deviations from the Hardy-Weinberg equilibrium. To evaluate the genetic association, we applied both dominant and recessive models, and the most appropriate model was selected based on their effect size and statistical significance.

We employed two analytical approaches: logistic regression and survival analysis. For logistic regression analysis, the characteristics between case and control groups were assessed using Fisher’s exact test or the chi-squared test. A multivariable logistic regression analysis that incorporated variables with *p*-values <0.05 from the univariate analysis, in addition to age, sex, and *ABCB1* rs3842, was performed. Unadjusted and adjusted odds ratios (ORs) with 95% confidence intervals (CIs) were calculated from the univariate and multivariable analyses. The fitness of the prediction model to the data was evaluated using the Hosmer–Lemeshow goodness-of-fit. The discriminative power of the model was assessed using the area under the receiver operating characteristic curve (AUROC). For survival analysis, we set the follow-up time as 1 year, and the time between the initiation of DOACs and the bleeding event was calculated in days. If a patient did not experience any bleeding event within the 1-year study period, he or she was censored. We used whichever came first between the last prescribed date or 365 days as the censored time. We employed Cox proportional hazard regression models and calculated the crude and adjusted hazard ratios (HRs) with 95% CIs.

To develop a risk scoring system, the adjusted OR (AOR) was divided by the minimum AOR observed within the variables and rounded to the closest integer. We subsequently conducted a logistic regression analysis to compare the observed and predicted risks. A *p*-value <0.05 was considered statistically significant. All the statistical analyses were performed using two-tailed statistics and SPSS version 20.0 (IBM Corp., Armonk, NY, United States).

## Results

**FIGURE 1:**
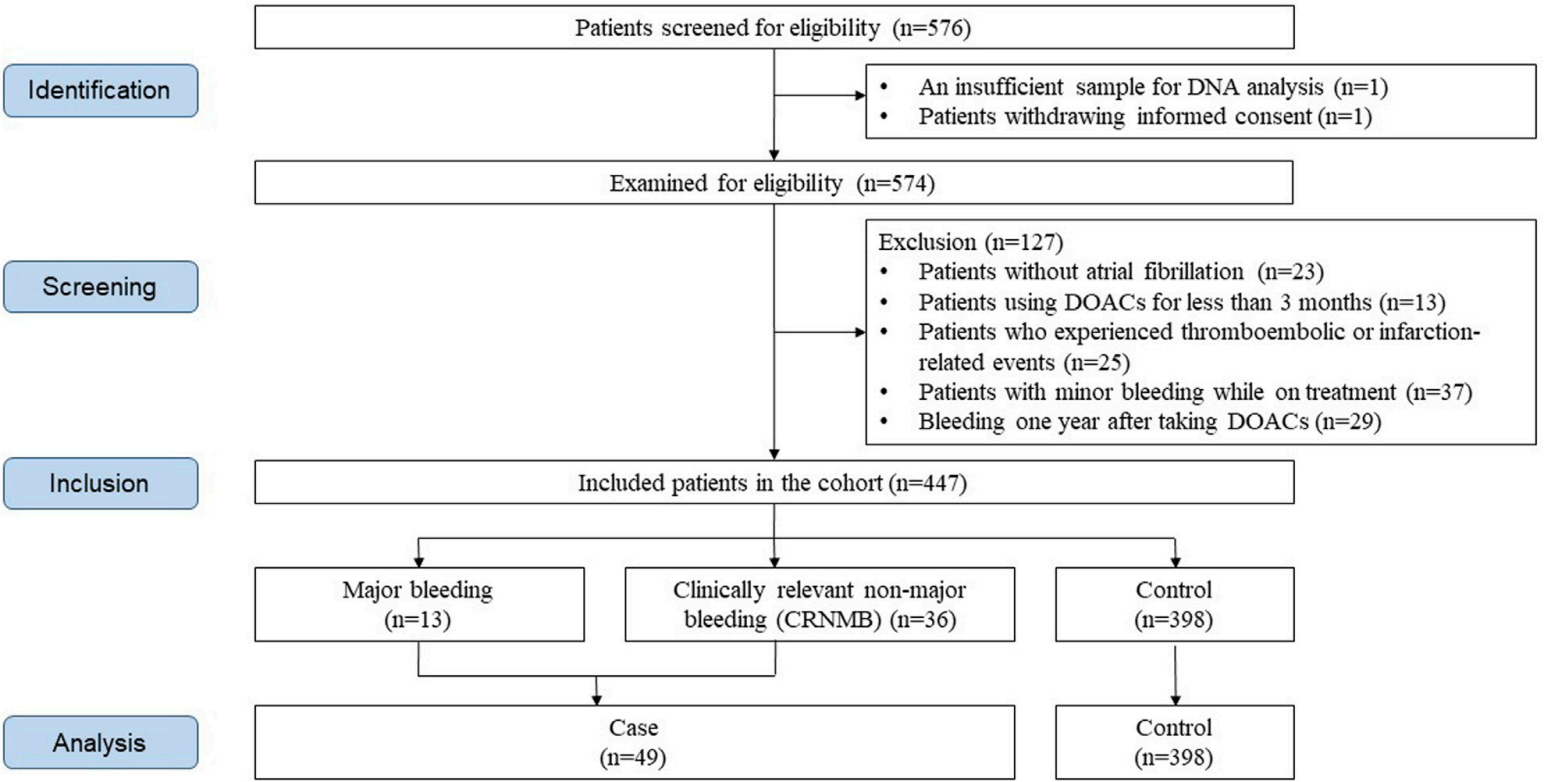
Flowchart of study patient selection.

Of the initial 576 enrolled patients, 447 were included in the final analysis after excluding those not meeting the criteria (Figure 1). Among them, 13 experienced major bleeding, and 36 patients had CRNMB. The demographic and clinical characteristics of the eligible patients are presented in Table 1. The mean age of the participants was 70 years, and 164 patients (36.7%) were female. Patients with severe renal impairment (CrCl less than 30 mL/min) were susceptible to bleeding compared to those with moderate or normal renal function (26.3% vs. 10.3%, *p* = 0.047). The types of DOACs exhibited a statistically significant difference between the bleeding and non-bleeding groups (*p* = 0.003). Of the 71 patients on rivaroxaban, 16 (22.5%) experienced a bleeding event, which had the highest incidence, followed by edoxaban (11.6%), apixaban (7.3%), and dabigatran (5.8%). The significant difference in the exposed dosage of DOACs was observed between the bleeding and non-bleeding group (*p* = 0.018). While 36.4% experienced bleeding in the overdosed group, 9.5% of the standard-dosed patients, and 12.0% of the underdosed patients experienced bleeding, respectively. Moreover, patients with anemia showed higher bleeding incidence than those without anemia (17.8% vs. 8.5%, *p* = 0.006).

**TABLE 1:** Baseline characteristics of patients who were administered direct oral anticoagulants.

Characteristic	Bleeding	No bleeding	*p*-value
	(n = 49)	(n = 398)	
Sex			0.994
Female	18 (36.7)	146 (36.7)	
Male	31 (63.3)	252 (63.3)	
Age (years)			0.889
<65	15 (30.6)	118 (29.6)	
≥65	34 (69.4)	280 (70.4)	
BMI (kg/m^2^)			0.429
<25	23 (48.9)	208 (55.0)	
≥25	24 (51.1)	170 (45.0)	
Creatinine clearance (mL/min)			0.047
<30	5 (10.6)	14 (3.7)	
≥30	42 (89.4)	365 (96.3)	
Types of DOACs			0.003
Apixaban	13 (26.5)	164 (41.2)	
Edoxaban	17 (34.7)	130 (32.7)	
Rivaroxaban	16 (32.7)	55 (13.8)	
Dabigatran	3 (6.1)	49 (12.3)	
Prescription dose^a^			0.018
Underdose	17 (34.7)	125 (31.4)	
Standard dose	28 (57.1)	266 (66.8)	
Overdose	4 (8.2)	7 (1.8)	
Co-medications			
Antiplatelets	3 (6.1)	51 (12.8)	0.175
ACEIs or ARBs	18 (36.7)	174 (43.7)	0.351
Beta-blockers	38 (77.6)	287 (72.1)	0.420
Calcium channel blockers	12 (24.5)	108 (27.1)	0.693
Diuretics	11 (22.4)	106 (26.6)	0.530
Statins	27 (55.1)	232 (58.3)	0.670
CYP inhibitors^b^	7 (14.3)	57 (14.4)	0.984
Previous myocardial infarction	4 (10.3)	35 (89.7)	1.000
Previous stroke/TIA/TE	27 (14.0)	166 (86.0)	0.074
Previous bleeding events	4 (18.2)	18 (81.8)	0.285
Comorbidities			
Hypertension	33 (67.3)	269 (67.6)	0.973
Diabetes mellitus	14 (28.6)	113 (28.4)	0.979
Congestive heart failure	5 (10.2)	76 (19.1)	0.127
Liver disease	0 (0)	9 (2.3)	0.606
Anemia	21 (42.9)	97 (24.4)	0.006
Smoking	10 (22.3)	89 (24.7)	0.830
Alcohol	17 (37.8)	133 (36.6)	0.881
CHA_2_DS_2_-VASc score			0.858
<3	17 (34.7)	133 (33.4)	
≥3	32 (65.3)	265 (66.6)	
Modified HAS-BLED score^c^			0.665
<3	33 (67.3)	280 (70.4)	
≥3	16 (32.7)	118 (29.6)	

ACEIs, angiotensin-converting enzyme inhibitors; ARBs, angiotensin II receptor blockers; BMI, body mass index; CYP, cytochrome P450 family; DOACs, direct oral anticoagulants; TE, thromboembolism; TIA, transient ischemic attack.

^a^ Standard dose was defined according to the FDA-approved labeling.

^b^ CYP inhibitors included amiodarone, dronedarone, diltiazem, verapamil, and imatinib.

^c^ The modified HAS-BLED score includes the following factors: hypertension, abnormal renal or liver function, stroke, bleeding history or predisposition, elderly (age ≥65 years), concomitant drug and alcohol use; range 0–8, excluding liable international normalized ratio (INR).

The grouped genotype analysis is shown in Table 2. The genotype distributions were all in Hardy-Weinberg equilibrium, suggesting the representativeness of all samples. Five SNPs of *RYR2*, specifically rs10925391, rs12594, rs17682073, rs3766871, and rs6678625, and *ABCB1* rs3842 showed a significant statistical difference between bleeding and non-bleeding groups. For *RYR2* polymorphisms, individuals with the CC genotype for rs10925391 and with the GG genotype for rs12594 experienced more bleeding events than those carrying the A allele (16.5% vs. 9.2%, *p* = 0.035; 35.3% vs. 9.9%, *p* = 0.006, respectively). Patients with the wild homozygotes of rs17682073 and rs3766871 had more bleeding than individuals with the variant allele (13.2% vs. 5.6%, *p* = 0.015; 12.4% vs. 3.0%, *p* = 0.023, respectively). Furthermore, patients carrying the T allele of rs6678625 tended to experience more bleeding events (27.3% vs. 9.7%, *p* = 0.006). In addition to *RYR2*, bleeding events occurred more often in patients with the *ABCB1* rs3842 CC genotype (21.7% vs. 9.8%, *p* = 0.014).

**TABLE 2:** Effects of gene polymorphisms on bleeding complications.

Gene polymorphism	Grouped genotypes	Bleeding	No bleeding	*p*-value
		(n = 49)	(n = 398)	
*RYR2*				
rs10925391 (A>C)	AA, AC	31 (63.3)	305 (77.0)	0.035
	CC	18 (36.7)	91 (23.0)	
rs12594 (A>G)	AA, AG	42 (87.5)	384 (97.2)	0.006
	GG	6 (12.5)	11 (2.8)	
rs2253273 (A>G)	AA, AG	17 (34.7)	99 (25.1)	0.148
	GG	32 (65.3)	296 (74.9)	
rs2256242 (A>G)	AA, AG	30 (61.2)	221 (55.7)	0.459
	GG	19 (38.8)	176 (44.3)	
rs17682073 (A>G)	AA	40 (83.3)	262 (66.0)	0.015
	AG, GG	8 (16.7)	135 (34.0)	
rs3765097 (C>T)	CC, CT	19 (39.6)	175 (44.1)	0.553
	TT	29 (60.4)	222 (55.9)	
rs3766871 (G>A)	GG	47 (95.9)	331 (83.6)	0.023
	GA, AA	2 (4.1)	65 (16.4)	
rs684923 (C>T)	CC, CT	22 (44.9)	150 (37.8)	0.334
	TT	27 (55.1)	247 (62.2)	
rs6678625 (C>T)	CC	40 (81.6)	373 (94.0)	0.006
	CT, TT	9 (18.4)	24 (6.0)	
rs2253831 (C>T)	CC, CT	28 (57.1)	181 (45.8)	0.134
	TT	21 (42.9)	214 (54.2)	
*ABCB1*				
rs3842 (T>C)	TT, TC	39 (79.6)	361 (90.9)	0.014
	CC	10 (20.4)	36 (9.1)	

**FIGURE 2:**
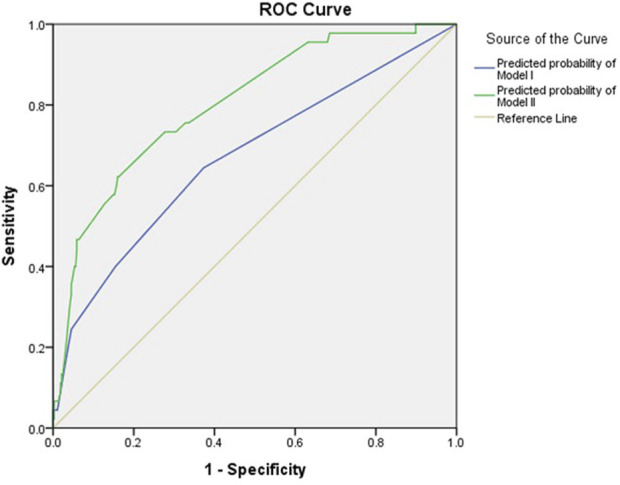
AUROC for bleeding using demographic and genetic factors. The blue line represents the predicted probability of Model I, while the green line represents that of Model II. The yellow line is the reference.

Multivariable regression analyses were conducted in two analytical approaches, incorporating clinical and genetic variables that were significant in the univariate analysis in addition to age and sex. For logistic regression analysis, two models were constructed: Model I included demographic actors only, and Model II included both demographic and genetic factors (Table 3). In Model I, rivaroxaban was associated with a 2.9-fold increased risk of bleeding (95% CI: 1.5–5.9) compared with other DOACs, and patients with anemia showed a 2.5-fold increased bleeding risk (95% CI: 1.3–4.8) compared with those without anemia after adjusting for covariates. When genetic factors were incorporated into Model I, a significant association with bleeding was observed for overdosing of DOACs and CrCl <30 mL/min in addition to the type of DOAC and anemia. Overdosed individuals displayed a 5.2-fold increased risk of bleeding (95% CI: 1.2–23.1), and patients with severely impaired renal function exhibited a 4.0-fold higher risk than those with a CrCl ≥30 mL/min (95% CI: 1.1–14.8). Among the genetic factors, the *RYR2* rs3766871 GG genotype had the most significant impact on bleeding risk, increasing the risk for GG genotype carriers by 6.6 times (95% CI: 1.4–32.2) compared with A allele carriers. Individuals with *RYR2* rs12594 variant homozygotes (GG) experienced a 5.9-fold increased bleeding risk (95% CI: 1.7–20.7) compared to those carrying the wild-type allele. As for *RYR2* rs17682073 and rs6678625, patients with the AA genotype of rs17682073 and the T allele of rs6678625 experienced a 3.5-fold (95% CI: 1.4–8.9) and a 3.7-fold (95% CI: 1.5–9.5) increased risk of bleeding, respectively. The Hosmer–Lemeshow test indicated good model fit for both models (χ2 = 0.734, *p* = 0.693 for Model I; χ2 = 4.950 and *p* = 0.550 for Model II). The AUROC of Model I was 0.670 (95% CI: 0.579–0.761), whereas that of Model II showed an increased AUROC of 0.803 (95% CI: 0.735–0.871), demonstrating enhanced performance upon incorporating genetic factors (Figure 2).

**TABLE 3:** Univariate and multivariable regression analyses to identify predictors for bleeding in patients treated with direct oral anticoagulants.

Predictors	Unadjusted OR (95% CI)	Model I		Model II	
		Adjusted OR (95% CI)	*p*-value	Adjusted OR (95% CI)	*p*-value
Age ≥65 years	0.96 (0.50–1.82)				
Female	1.00 (0.54–1.86)				
Overdose	4.96 (1.40–17.62)	3.84 (0.98–15.01)	0.053	5.17 (1.16–23.05)	0.031
Rivaroxaban	3.02 (1.56–5.86)	2.92 (1.46–5.85)	0.002	3.90 (1.83–8.28)	<0.001
Anemia	2.33 (1.26–4.29)	2.53 (1.34–4.79)	0.004	2.37 (1.17–4.80)	0.017
CrCl <30 mL/min	3.11 (1.06–9.01)			3.96 (1.06–14.83)	0.041
*ABCB1* rs3842 CC	2.57 (1.19–5.58)				
*RYR2* rs10925391 CC	1.95 (1.04–3.64)				
*RYR2* rs12594 GG	4.98 (1.76–14.17)			5.89 (1.67–20.74)	0.006
*RYR2* rs17682073 AA	2.58 (1.17–5.65)			3.51 (1.39–8.90)	0.008
*RYR2* rs3766871 GG	4.55 (1.09–19.61)			6.64 (1.37–32.24)	0.019
*RYR2* rs6678625 T allele	3.50 (1.52–8.04)			3.72 (1.46–9.49)	0.006

CI, confidence interval; CrCl, creatinine clearance; OR, odds ratio. Model I included demographic variables, including age, sex, overdose, rivaroxaban, anemia, and CrCl <30 mL/min. Model II, included *ABCB1* rs3842 and *RYR2* rs10925391, rs12594, rs17682073, rs3766871, and rs6678625 in addition to clinical variables from Model I.

The risks of clinical characteristics and gene polymorphisms for bleeding complications were assessed in the survival analysis, as presented in Supplementary Tables S1, S2. Among these variables, those with a significant *p*-value were incorporated into the multivariable regression analysis (Supplementary Table S3). Rivaroxaban demonstrated a 5.9-fold higher risk of bleeding (95% CI: 1.6–22.1) than dabigatran. Additionally, overdosed individuals showed a 7.5-fold increase in bleeding risk (95% CI: 2.1–27.3) compared to underdosed patients. Among comorbidities, patients with anemia and severe renal impairment exhibited a 3.0-fold (95% CI: 1.6–5.8) and a 3.7-fold (95% CI: 1.3–10.9) higher risk of bleeding than those without these clinical conditions, respectively. For *RYR2* polymorphisms, patients carrying the *RYR2* rs12594 GG genotype had approximately three times the incidence of bleeding than those with the A allele (95% CI: 1.1–8.3). Individuals with rs17682073 AA genotype and the rs3766871 GG genotype showed a 3.2-fold (95% CI: 1.4–7.5) and a 6.7-fold (95% CI: 1.5–28.8) increased bleeding risks compared to variant allele carriers, respectively. Furthermore, *RYR2* rs6678625T allele carriers were more likely to experience bleeding than those with the CC genotype (adjusted HR: 2.9, 95% CI: 1.3–6.3).

**FIGURE 3:**
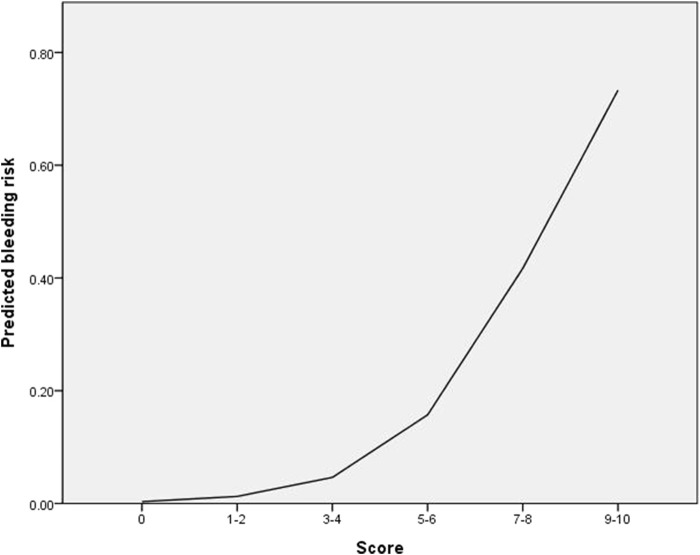
Logistic regression curve of the probability of bleeding risk.

Using the significant variables in Model II, a risk scoring system was constructed: the AOR of these variables was divided by the AOR of anemia, which had the smallest value. Consequently, the following points were assigned: overdose (2 points), rivaroxaban (2 points), anemia (1 point), CrCl <30 mL/min (2 points), *RYR2* rs12594GG (2 points), rs17682073 AA (1 point), rs6678625 T allele (2 points), and rs3766871GG (3 points). The risk score ranged from 0 to 15, but none of the patients had scores over 11. The risk estimates obtained from the logistic regression curve are depicted in Figure 3. For patients with 0, 1–2, 3–4, 5–6, 7–8, and 9–10 points, the predicted risk of bleeding was 0%, 1.2%, 4.6%, 15.7%, 41.7%, and 73.3%, respectively (Table 4). The AUROC of this logistic regression curve was 0.768 (95% CI: 0.690–0.846, *p* < 0.001).

**TABLE 4:** The observed and predicted bleeding risk (%) by risk scoring system.

Score	Bleeding (n)	Total (n)	Observed bleeding risk (%)	Predicted bleeding risk (%)
0	0	13	0.0	0.0
1–2	1	34	2.9	1.2
3–4	10	220	4.5	4.6
5–6	17	117	14.5	15.7
7–8	15	33	45.5	41.7
9–10	2	3	66.7	73.3

## Discussion

Previous studies have demonstrated an association between *RYR2* polymorphisms and arrhythmias; however, no study has evaluated bleeding complications. The findings of this study indicated that severe renal impairment (CrCl <30 mL/min), anemia, overdosing of DOACs, and rivaroxaban use significantly increased the bleeding risk. Of genetic factors, *RYR2* rs12594, rs17682073, rs3766871, and rs6678625 were associated with bleeding complications. The risk scoring system that was constructed with these variables satisfactorily predicted bleeding risk, achieving an AUROC of 0.768.

RyRs mediate the release of Ca^2+^ from the endoplasmic and sarcoplasmic reticulum (SR) into the cytosol and convert different extracellular stimuli into intracellular Ca^2+^ signals, thereby playing a critical role in muscle contraction (Sun and Xu, 2019). Three *RYR* genes have been identified: *RYR1*, *RYR2*, and *RYR3*. The *RYR1* gene (on human chromosome 19q13.1), which is responsible for Ca^2+^ release in skeletal muscle, has been associated with disorders such as King-Denborough syndrome, rhabdomyolysis-myalgia syndrome, and bleeding abnormalities (Dowling et al., 2011; Lopez et al., 2016; Witting et al., 2018). *RYR3* (on 15q13.3-14), which is differentially expressed in the brain, is the least understood isoform of *RYR*s, and no disease has been conclusively associated with *RYR3* mutations. A recent case report has suggested that variants in *RYR3* may potentially be linked to nemaline myopathy, but further research is still needed to establish this connection (Nilipour et al., 2018).

*RYR2* gene (on 1q42.1-43) encodes the cardiac Ca^2+^ channel, and mutations have been linked to catecholaminergic polymorphic ventricular tachycardia (CPVT) and SCD (Napolitano and Priori, 2007; Steinberg et al., 2023). In particular, the *RYR2* rs3766871, one of the most extensively studied polymorphisms, is a missense variant that leads to the substitution of a serine residue with glycine (Zhang et al., 2003). This substitution causes destabilization of the channel and phosphorylation of protein kinase C, resulting in abnormal spontaneous leaks of Ca^2+^ from the SR (Milting et al., 2006; Koop et al., 2008). Due to this effect, *RYR2* rs3766871 has been investigated in relation to ventricular arrhythmias and SCD in chronic heart failure (Ran et al., 2010; Francia et al., 2015). Francia et al. (2015) demonstrated that the variant allele increased susceptibility to ventricular tachycardia and ventricular fibrillation in patients with heart failure (HR: 3.49; 95% CI: 1.14–10.62). A Chinese cohort study of 1,244 patients with chronic heart failure indicated that the variant allele of rs3766871 in *RYR2* was an independent risk factor that increased the risk of cardiac death (HR: 1.92; 95% CI: 1.24–2.94) and ventricular arrhythmias (OR: 1.66; 95% CI: 1.21–2.26) (Ran et al., 2010). Considering that arrhythmia is recognized as a risk factor for stroke, our finding is explicable to some extent.

*RYR2* rs12594 is located in the 3′-untranslated region (3′-UTR) of *RYR2*. Despite being a non-coding part of mRNA, SNPs in the 3′-UTR can influence gene expression through mechanisms such as mRNA degradation, translation, and localization (Mayr, 2017). While its role in bleeding or cardiovascular diseases remains incompletely understood, research has explored its implications in cancer. Chen et al. (2019) observed that the A allele of *RYR2* rs12594 was associated with overall survival of patients with astrocytoma, demonstrating the impact of *RYR2* on the prognosis of lower-grade brain gliomas. Another case-control study showed a relationship between rs12594 polymorphism and the risk of breast cancer in a Chinese population. The *RYR2* rs12594GG genotype was related to decreased breast cancer risk even after being adjusted by age, estrogen receptor status, progesterone receptor status, menopausal status, tumor size, and tumor stage (Wei et al., 2020). Thus, the G allele of rs12594 may contribute to a change in RYR2 expression, thereby increasing susceptibility to bleeding. However, further studies are required due to limited research on cardiovascular diseases.

*RYR2* rs6678625 and rs17682073 were associated with an approximately three-fold increased bleeding risk in our study. Despite being intron variants, these SNPs have the potential to influence mRNA splicing, leading to alterations in protein expression or activity (Pagani and Baralle, 2004; Raponi and Baralle, 2010). According to the eQTL analysis by GTEx, the variant allele of rs6678625 had lower RYR2 expression in the aorta and coronary artery (*p* = 2.6 × 10^−6^ and 2.8 × 10^^−4^, respectively) (GTEx Consortium, 2017). Westaway et al. (2011) employed a systematic candidate-gene approach to identify genes linked to SCD risk in a Caucasian population with coronary artery diseases. The findings revealed that *RYR2* rs6678625 was one of the SNPs that was significantly associated with SCD, but it lost significance in the replication sample. Based on these findings, it could be speculated that polymorphism of this SNP could potentially alter the expression or activity of RYR2, thereby leading to an elevated risk of bleeding. According to a previous study, *RYR2* rs17682073 was identified as one of the SNPs associated with non-autism spectrum disorders in Central European populations (Skafidas et al., 2014); however, the available studies are limited, necessitating additional research.

DOACs are primarily excreted through the kidneys, with excretion rates ranging from 27% to 80% (Steffel et al., 2021). In case of rivaroxaban, the area under the curve increased by 44% in individuals with mild renal impairment (CrCl 50–75 mL/min) and by 64% in those with severe impairment (Kubitza et al., 2010). Therefore, adjusting the dosage of DOACs based on kidney function is imperative. However, there is limited available data on patients with severe chronic kidney disease since individuals with a CrCl <30 mL/min were excluded from all landmark trials of DOACs. Our findings revealed that despite dose adjustments based on kidney function, severe renal impairment remained a significant risk factor for bleeding. A study conducted by Yao et al. (2017) demonstrated a 1.3-fold (95% CI: 1.0–1.6) increased bleeding risk in individuals with moderate to severe renal impairment which is consistent with the findings of our study.

Patients who were prescribed an excessive dose of DOACs showed a five-fold increased bleeding risk according to the findings of our study. This elevated risk of bleeding was associated with increased plasma concentrations resulting of DOACs from reduced excretion and increased dosage. The higher concentrations can lead to more potent pharmacodynamic effects of DOACs, further amplifying the risk of bleeding. Given the increased susceptibility to bleeding risks associated with anticoagulation observed in Asian populations (Gaikwad et al., 2014), it is essential to implement closer monitoring for patients with impaired renal function in order to prevent overdosing of DOACs.

Rivaroxaban was the least safe DOAC with an approximately 4-fold higher risk of bleeding than other DOACs in our study. In a meta-analysis that compared the safety of apixaban and rivaroxaban using real-world data, those treated with apixaban exhibited a reduced risk of major bleeding and CRNMB (relative risk (RR) 0.73, 95% CI: 0.58–0.93 and RR 0.59, 95% CI: 0.50–0.70, respectively) (Aryal et al., 2019). Regarding this result, the safety profile including its PK characteristics of rivaroxaban has been elucidated. As rivaroxaban is given as a once daily dose, the peak concentration is higher than that of other DOACs. For example, the peak-to-trough ratio of rivaroxaban is approximately 10 (at a dose of 10–20 mg once daily), whereas for apixaban, it is approximately 3 (at a dose of 5 mg twice daily) (Aryal et al., 2019). This discrepancy explains the increased incidence of bleeding complications associated with rivaroxaban observed in our study.

Anemia, which refers to low levels of healthy red blood cells or hemoglobin, was associated with increased bleeding in our study. Red blood cells contribute not only to transporting oxygen but also to initiating hemostasis together with platelets and to stabilizing fibrin and clot structure (Lassila and Weisel, 2023). Their role has made anemia an independent risk factor for major bleeding during anticoagulant therapy. In the RE-LY trial with 18,113 randomized patients with AF, anemia was related to major bleeding complications (HR 2.14, 95% CI: 1.87–2.46) and discontinuation of anticoagulants (Westenbrink et al., 2015). In addition, a meta-analysis demonstrated that anemia was associated with a 78% increased risk of major bleeding (HR 1.78, 95% CI: 1.54–2.05) and a 77% increased risk of gastrointestinal bleeding (HR 1.77, 95% CI: 1.23–2.55) (Tu et al., 2021). This explains why many existing bleeding assessment tools incorporate anemia as a constituent factor within their scoring systems (Apostolakis et al., 2012).

Since bleeding is the most undesirable complication in patients taking anticoagulants, many risk scoring systems have been developed; however, most of them are applicable to vitamin K antagonist therapy (Shoeb and Fang, 2013). Among them, the CHA_2_DS_2_-VASc score, which was initially designed to predict the risk of stroke, exhibited effective prediction capability for major bleeding in individuals being treated with DOACs, with an AUROC of 0.68 (Yao et al., 2017). However, our risk scoring system that encompasses both clinical and genetic variables showed better prediction ability with an AUROC of 0.80. Considering that there was no significant difference in the CHA_2_DS_2_-VASc score or HAS-BLED score between the two groups in their baseline characteristics, genetic factors might considerably influence the onset of bleeding.

This study has a few limitations. While we collected samples prospectively, the risk of bias is associated with the retrospective patient data collection. Furthermore, the inclusion of only Asian participants and the relatively small sample size potentially restrict the generalization of the findings. We did not use multiple test correction methods like Bonferroni correction, often considered excessively conservative for a hypothesis-generating study, to prevent the potential loss of true positives. In addition, drug-gene interactions were not estimated. Given this limitation, careful interpretation is warranted when applying this risk scoring system in clinical settings. Therefore, further extensive cohort studies can validate our findings, thereby contributing to a deeper comprehension of the functional consequences of these polymorphisms. Nevertheless, the implication of a novel risk scoring system that incorporates genetic factors would help to assess susceptibility to bleeding, guide therapeutic interventions, and facilitate personalized medicine approaches.

## Conclusion

This study elucidated the association between *RYR2* gene polymorphisms and bleeding complications among patients treated with DOACs. After validation of the results, these findings and the constructed risk scoring system may help to predict the bleeding risk for such patients, thereby advocating for individualized DOAC treatment.

## Data Availability

The original contributions presented in the study are included in the article/Supplementary Material, further inquiries can be directed to the corresponding authors.
