# Supplementary material for: Construction of a risk scoring system using clinical factors and RYR2 polymorphisms for bleeding complications in patients on direct oral anticoagulants
Source: Front Pharmacol. 2023 Nov 15;14:1290785. doi: 10.3389/fphar.2023.1290785 (PMC10684747; doi:10.3389/fphar.2023.1290785)
Supplement: Supplementary file 1 [file Table1.docx]

Supplementary Table 1. Effects of baseline characteristics on bleeding in study participants assessed using survival analysis.

| Characteristic | Bleeding  (n = 49) | No bleeding  (n = 427) | Crude HR  (95% CI) | *p*-value |
| --- | --- | --- | --- | --- |
| Sex |  |  |  | 0.986 |
| Male | 31 (63.3) | 270 (63.2) | 1 |  |
| Female | 18 (36.7) | 157 (36.8) | 0.99 (0.56–1.78) |  |
| Age (years) |  |  |  | 0.735 |
| < 65 | 15 (30.6) | 126 (29.5) | 1 |  |
| ≥ 65 | 34 (69.4) | 301 (70.5) | 0.90 (0.48–1.67) |  |
| BMI (kg/m^2^) |  |  |  | 0.396 |
| < 25 | 23 (48.9) | 223 (54.9) | 1 |  |
| ≥ 25 | 24 (51.1) | 183 (45.1) | 1.27 (0.72–2.24) |  |
| Creatinine clearance (mL/min) |  |  |  | 0.021 |
| < 30 | 5 (10.6) | 15 (3.7) | 2.84 (1.13–7.19) |  |
| ≥ 30 | 42 (89.4) | 392 (96.3) | 1 |  |
| Types of DOACs |  |  |  | 0.011 |
| Dabigatran | 3 (6.1) | 50 (11.7) | 1 |  |
| Apixaban | 13 (26.5) | 171 (40.0) | 1.26 (0.36–4.41) |  |
| Edoxaban | 17 (34.7) | 141 (33.0) | 1.92 (0.56–6.56) |  |
| Rivaroxaban | 16 (32.7) | 65 (15.2) | 3.69 (1.08–12.67) |  |
| Prescription dose ^a^ |  |  |  | 0.034 |
| Underdose | 17 (34.7) | 137 (32.1) | 1 |  |
| Standard dose | 28 (57.1) | 281 (65.8) | 0.83 (0.46–1.52) |  |
| Overdose | 4 (8.2) | 9 (2.1) | 3.08 (1.04–9.14) |  |
| Co-medications |  |  |  |  |
| Antiplatelets | 3 (6.1) | 55 (12.9) | 0.46 (0.20–1.07) | 0.181 |
| ACEIs or ARBs | 18 (36.7) | 188 (44.0) | 0.73 (0.42–1.28) | 0.287 |
| Beta-blockers | 38 (77.6) | 287 (71.4) | 1.34 (0.72–2.49) | 0.396 |
| Calcium channel blockers | 12 (24.5) | 108 (26.2) | 0.94 (0.50–1.79) | 0.857 |
| Diuretics | 11 (22.4) | 114 (26.7) | 0.79 (0.42–1.47) | 0.490 |
| Statins | 27 (55.1) | 248 (58.1) | 0.85 (0.48–1.51) | 0.579 |
| CYP inhibitors ^b^ | 7 (14.3) | 59 (13.9) | 1.06 (0.47–2.40) | 0.889 |
| Previous myocardial infarction | 4 (10.3) | 38 (8.9) | 0.87 (0.33–2.29) | 0.791 |
| Previous stroke/TIA/TE | 27 (14.0) | 180 (42.2) | 1.55 (0.88–2.72) | 0.124 |
| Previous bleeding events | 4 (18.2) | 18 (4.2) | 1.89 (0.49–7.33) | 0.213 |
| Comorbidities |  |  |  |  |
| Hypertension | 33 (67.3) | 290 (67.9) | 0.98 (0.54–1.79) | 0.949 |
| Diabetes mellitus | 14 (28.6) | 123 (28.8) | 0.99 (0.54–1.85) | 0.984 |
| Congestive heart failure | 5 (10.2) | 84 (19.7) | 0.48 (0.24–0.97) | 0.110 |
| Liver disease | 0 (0) | 9 (2.1) | - | 0.328 |
| Anemia | 21 (42.9) | 104 (24.4) | 2.24 (1.27–3.95) | 0.004 |
| Smoking | 10 (22.3) | 134 (31.4) | 0.88 (0.45–1.73) | 0.726 |
| Alcohol | 17 (37.8) | 137 (35.0) | 1.10 (0.61–2.01) | 0.742 |
| CHA_2_DS_2_-VASc score |  |  |  | 0.605 |
| < 3 | 17 (34.7) | 139 (32.6) | 1 |  |
| ≥ 3 | 32 (65.3) | 288 (67.4) | 0.86 (0.47–1.57) |  |
| Modified HAS-BLED score ^c^ |  |  |  | 0.751 |
| < 3 | 33 (67.3) | 300 (70.3) | 1 |  |
| ≥ 3 | 16 (32.7) | 127 (29.7) | 1.10 (0.60–2.02) |  |

ACEIs, angiotensin converting enzyme inhibitors; ARBs, angiotensin II receptor blockers; BMI, body mass index; CYP, cytochrome P450 family; DOACs, direct oral anticoagulants; HR: hazard ratio; TE: thromboembolism; TIA, transient ischemic attack.

^a^ Standard dose was defined according to the FDA-approved labeling.

^b^ CYP inhibitors included amiodarone, dronedarone, diltiazem, verapamil, and imatinib.

^c^ The modified HAS-BLED score includes the following factors; hypertension, abnormal renal or liver function, stroke, bleeding history or predisposition, elderly (age ≥ 65 years), concomitant drug and alcohol use; range 0–8, excluding liable international normalized ratio (INR)

Supplementary Table 2. Risk of bleeding complications associated with gene polymorphisms in survival analysis.

| Gene polymorphism | Grouped genotypes | Bleeding  (n = 49) | No bleeding  (n = 427) | Crude HR  (95% CI) | *p*-value |
| --- | --- | --- | --- | --- | --- |
| *RYR2* |  |  |  |  |  |
| rs10925391 (A>C) | AA, AC | 31 (63.3) | 328 (77.4) | 1 | 0.034 |
|  | CC | 18 (36.7) | 96 (22.6) | 1.86 (1.04 – 3.32) |  |
| rs12594 (A>G) | AA, AG | 42 (87.5) | 410 (96.7) | 1 | 0.002 |
|  | GG | 6 (12.5) | 14 (3.3) | 3.59 (1.53 – 8.44) |  |
| rs2253273 (A>G) | AA, AG | 17 (34.7) | 111 (26.2) | 1.44 (0.80 – 2.60) | 0.219 |
|  | GG | 32 (65.3) | 313 (73.8) | 1 |  |
| rs2256242 (A>G) | AA, AG | 30 (61.2) | 240 (56.3) | 1.22 (0.69 – 2.17) | 0.492 |
|  | GG | 19 (38.8) | 186 (43.7) | 1 |  |
| rs17682073 (A>G) | AA | 40 (83.3) | 285 (66.9) | 2.38 (1.11 – 5.07) | 0.021 |
|  | AG, GG | 8 (16.7) | 141 (33.1) | 1 |  |
| rs3765097 (C>T) | CC, CT | 19 (39.6) | 185 (43.4) | 1 | 0.561 |
|  | TT | 29 (60.4) | 241 (56.6) | 1.19 (0.67 – 2.10) |  |
| rs3766871 (G>A) | GG | 47 (95.9) | 356 (83.8) | 4.32 (1.05 – 17.79) | 0.027 |
|  | GA, AA | 2 (4.1) | 69 (16.2) | 1 |  |
| rs684923 (C>T) | CC, CT | 22 (44.9) | 163 (38.3) | 1.31 (0.75 – 2.30) | 0.349 |
|  | TT | 27 (55.1) | 263 (61.7) | 1 |  |
| rs6678625 (C>T) | CC | 40 (81.6) | 398 (93.4) | 1 | 0.002 |
|  | CT, TT | 9 (18.4) | 28 (6.6) | 3.03 (1.47 – 6.25) |  |
| rs2253831 (C>T) | CC, CT | 28 (57.1) | 195 (46.0) | 1.53 (0.87 – 2.70) | 0.136 |
|  | TT | 21 (42.9) | 229 (54.0) | 1 |  |
| *ABCB1* |  |  |  |  |  |
| rs3842 (T>C) | TT, TC | 39 (79.6) | 388 (91.1) | 1 | 0.009 |
|  | CC | 10 (20.4) | 38 (8.9) | 2.45 (1.23 – 4.92) |  |

HR: hazard ratio

Supplementary Table 3. Cox proportional hazard regression analysis for identifying predictors of bleeding in patients treated with direct oral anticoagulants.

| **Predictors** | **Unadjusted HR**  **(95% CI)** | **Adjusted HR**  **(95% CI)** | ***p*-value** |
| --- | --- | --- | --- |
| Age ≥ 65 years | 0.90 (0.48–1.67) | 0.53 (0.27–1.04) | 0.065 |
| Female | 0.99 (0.56–1.78) |  |  |
| Prescription dose |  |  |  |
| Underdose | 1 | 1 |  |
| Standard dose | 0.83 (0.46–1.52) | 1.89 (0.90–3.98) | 0.093 |
| Overdose | 3.08 (1.04–9.14) | 7.51 (2.06–27.33) | 0.002 |
| Types of DOACs |  |  |  |
| Dabigatran | 1 | 1 |  |
| Apixaban | 1.26 (0.36–4.41) | 1.35 (0.37–4.95) | 0.648 |
| Edoxaban | 1.92 (0.56–6.56) | 1.44 (0.40–5.25) | 0.580 |
| Rivaroxaban | 3.69 (1.08–12.67) | 5.94 (1.60–22.12) | 0.008 |
| Anemia | 2.24 (1.27–3.95) | 3.03 (1.59–5.75) | 0.001 |
| CrCl < 30 mL/min | 2.84 (1.13–7.19) | 3.73 (1.28–10.88) | 0.016 |
| *ABCB1* rs3842 CC | 2.45 (1.23–4.92) |  |  |
| *RYR2* rs10925391 CC | 1.86 (1.04–3.32) |  |  |
| *RYR2* rs12594 GG | 3.59 (1.53–8.44) | 2.97 (1.06–8.31) | 0.038 |
| *RYR2* rs17682073 AA | 2.38 (1.11–5.07) | 3.22 (1.38–7.48) | 0.007 |
| *RYR2* rs3766871 GG | 4.32 (1.05–17.79) | 6.65 (1.53–28.81) | 0.011 |
| *RYR2* rs6678625 T allele | 3.03 (1.47–6.25) | 2.85 (1.30–6.26) | 0.009 |

CI: confidence interval; CrCl: creatinine clearance; HR: hazard ratio. Clinical and genetic variables that were significant in the univariate analysis in addition to age and sex were incorporated into the multivariable analysis.
